# Rab GTPases: Emerging Oncogenes and Tumor Suppressive Regulators for the Editing of Survival Pathways in Cancer

**DOI:** 10.3390/cancers12020259

**Published:** 2020-01-21

**Authors:** Priya D. Gopal Krishnan, Emily Golden, Eleanor A. Woodward, Nathan J. Pavlos, Pilar Blancafort

**Affiliations:** 1 Cancer Epigenetics Laboratory, The Harry Perkins Institute of Medical Research, 6 Verdun Street, Nedlands, WA 6009, Australia; priya.gopalkrishnan@research.uwa.edu.au (P.D.G.K.); emily.golden@uwa.edu.au (E.G.); eleanor.woodward@uwa.edu.au (E.A.W.); 2 School of Human Sciences, Faculty of Science, The University of Western Australia, 35 Stirling Highway Perth, Perth, WA 6009, Australia; 3 School of Biomedical Sciences, The University of Western Australia, Nedlands, WA 6009, Australia; nathan.pavlos@uwa.edu.au

**Keywords:** apoptosis, Rab GTPase, cancer, PI3K-AKT-mTOR

## Abstract

The Rab GTPase family of proteins are mediators of membrane trafficking, conferring identity to the cell membranes. Recently, Rab and Rab-associated factors have been recognized as major regulators of the intracellular positioning and activity of signaling pathways regulating cell growth, survival and programmed cell death or apoptosis. Membrane trafficking mediated by Rab proteins is controlled by intracellular localization of Rab proteins, Rab-membrane interactions and GTP-activation processes. Aberrant expression of Rab proteins has been reported in multiple cancers such as lung, brain and breast malignancies. Mutations in Rab-coding genes and/or post-translational modifications in their protein products disrupt the cellular vesicle trafficking network modulating tumorigenic potential, cellular migration and metastatic behavior. Conversely, Rabs also act as tumor suppressive factors inducing apoptosis and inhibiting angiogenesis. Deconstructing the signaling mechanisms modulated by Rab proteins during apoptosis could unveil underlying molecular mechanisms that may be exploited therapeutically to selectively target malignant cells.

## 1. Introduction

Cancers originate through dysregulation of normal cellular processes that either promote the growth, proliferation or migration of cells and/or suppress anti-tumorigenic functions such as programmed cell death or apoptosis. Intracellular communication and interaction of proteins within cellular components is fundamental for cell viability. These processes are regulated through multiple coordinated signaling cascades, which are orchestrated by a variety of proteins, enzymes and cellular receptors. Among the multiple signaling pathways, the mitogen-activated protein kinase (MAPK) and the phosphatidylinositol-3-kinase (PI3K) pathway play key roles in promoting cell survival and inhibiting apoptosis [1]. Vesicle trafficking is an emerging fundamental process enabling the transduction of these signals and transport of cargo between the specialized membrane-delimited intra-cellular compartments. Among the many small monomeric G proteins, Ras-associated binding (Rab) proteins are master regulators of vesicle trafficking and as such control a multitude of signaling cascades and biological processes [2]. Rab proteins confer spatial–temporal identity to intracellular membranes, therefore positioning signaling information in cells and tissues. Dysregulated expression of Rab-coding genes disrupts membrane trafficking and affects the regulation of multiple signaling pathways. Rab proteins influence cellular physiology, and differential regulation of Rab proteins play driving roles in diseases such as cancer, Alzheimer’s disease, and several other genetic disorders [3,4,5,6]. Regulating and normalizing Rab activity in many human diseases, such as cancer (“Rab-mediated editing”), represents an attractive new therapeutic avenue in molecular cancer therapeutics to precisely target neoplastic cells. Among the multiple Rabs discovered in humans known to regulate signaling pathways, many control cellular survival and apoptosis. This review provides an overview of Rab family members, insights into the regulation of apoptotic programs by Rabs and their role in human diseases associated with poor outcomes, such as Alzheimer’s and neoplastic diseases.

## 2. Structure and Function of Rab Proteins

**Figure 1:**
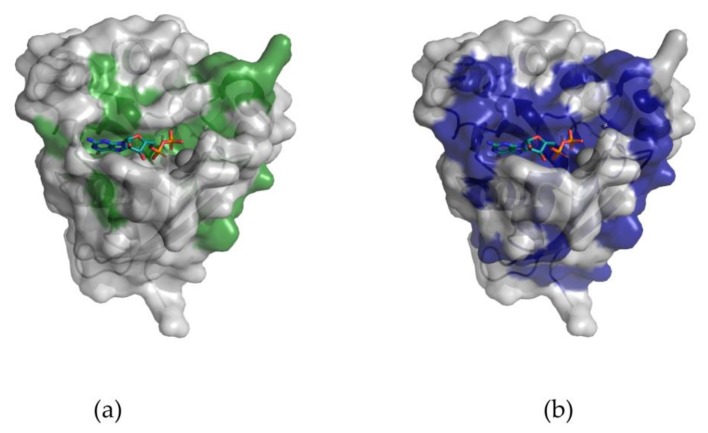
Evolutionary conservation of Rab GTPase proteins. (**a**) The surface representation of Rab1a crystal structure (green) from *Homo sapiens* (PDB ID: 4FML) shows residues that are fully conserved across all human Rab proteins (PDB IDs: 3TKL, 6IF2, 5LPM, 2IL1, 1X3S, 1Z0F, 2A5J, 2P5S, 6HUF, 1YZT, 2FG5, 1Z22, 4QXA, 2F7S, 20CB, 3TS0); (**b**) Residues that are 100% conserved across Rab1a proteins from multiple species (mouse, rat, wolf, human, pig, thale cress, slime mold, great pond snail) are shown in blue.

**Figure 2:**
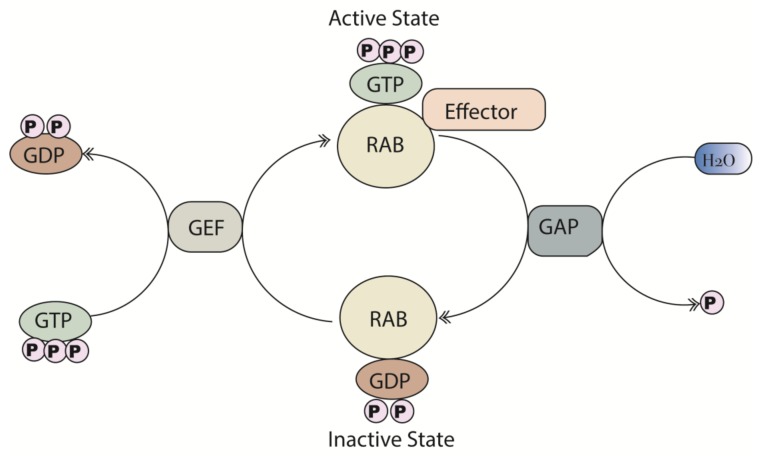
Schematic representation of the Rab GTPase cycle. Rab proteins oscillate between an active guanosine triphosphate (GTP)-bound state and an inactive guanosine diphosphate (GDP)-bound state. The activation and inactivation is regulated by guanine nucleotide exchange factors (GEFs) and GTPase-activating proteins (GAPs), respectively. Rab proteins, in the active (GTP-bound) state promote downstream signaling through the interaction of effector proteins.

Rab proteins constitute the largest family of the RAS superfamily of small GTPases with more than 60 members identified in humans [7,8]. Rab proteins are master regulators of vesicular transport, providing a molecular identity to specialized cellular membrane compartments. Given the large number and unique localization of Rab proteins, they share remarkable structural and sequence similarity (Figure 1a). Rab protein sequences are highly evolutionarily conserved across species with 100% identity between mammalian Rab1a and approximately 50% identity with Rab1a from *Arabidopsis thaliana* (Figure 1b), highlighting their fundamental role in cell physiology. There is particularly high conservation in the nucleotide-binding pocket, reflecting the main biochemical function, which is the hydrolysis of guanosine triphosphate (GTP) to guanosine diphosphate (GDP). This enables Rab proteins to act as molecular “on/off” switches as they oscillate between a GTP-bound (active) state and a GDP-bound (inactive) state [9]. Guanine nucleotide exchange factors (GEFs) and GTPase-activating proteins (GAPs) regulate this cycle of activation and deactivation. GEFs catalyze the exchange of GDP for a GTP molecule, activating the small GTPases [10]. Conversely, GAPs promote Rab inactivation by providing a catalytic group to accelerate the slow intrinsic GTP hydrolysis rate of the Rab-GTPases (Figure 2). Rab proteins, in the activated state (GTP-bound) promote downstream signaling by interacting with various effector proteins that function in specific stages of vesicular transport (ranging from membrane budding to fusion). Cells with dysregulated Rab expression, as a result of gene mutations and/or post-translational modifications such as prenylation and phosphorylation, which are essential for the proper functioning of Rabs, exhibit distinct variations in biological functionality [11].

## 3. Dysregulated Rab Expression in Cancer and other Genetic Diseases 

Digressive expression of Rabs has been implicated in multiple varied diseases and thus manifests as a wide range of severe effects. For example, Rab mutations are associated with genetic diseases including rare autosomal pleiotropic recessive disorders such as Griscelli syndrome, which affects both brain and immune system function, and Carpenter Syndrome, a developmental disorder characterized by inappropriate fusion of the skull during development. Griscelli syndrome is caused by a loss of function mutation in Rab27a, altering cytotoxic T-cell exocytosis and thus causing dysregulation of immune homeostasis, while Rab23 is mutated in Carpenter syndrome possibly resulting in dysregulation of Hedgehog signaling [3,12,13]. Furthermore, dysregulation of endocytosis is an early phenotype observed in Alzheimer’s disease. Endocytosis is sequentially regulated by Rab5 (in early endosomes) and Rab7a (in late endosomes), and both Rab proteins are upregulated in the brains of individuals with Alzheimer’s disease [14]. Beyond neurodegenerative disorders, recent studies identified Rab5b as a key regulator of hepatitis B virus production by controlling trafficking of the viral envelope from the endoplasmic reticulum to the multi-vesicular body [15].

In cancer, Rab proteins can either promote and/or suppress tumor growth and development. The majority of Rab genes are associated with the former, by acting as oncogenic drivers in a wide range of cancers. Amplification rather than mutation of Rab genes are generally associated with tumorigenesis and cancer progression as overexpression of these Rabs can activate growth and survival signaling pathways. For example, Rab1a overexpression in colorectal cancers is correlated with the mammalian target of rapamycin complex 1 (mTORC1) activation in tumors and this occurs through a direct interaction between Rab1a and mTORC1 [16]. Rab1a-mediated trafficking also affects the migration of cells through the trafficking of β1 integrins to the plasma membrane and localization to lipid rafts [17].

Similarly, Rab3d is overexpressed in a range of tumors including breast and lung cancers and correlates with increased metastatic behavior. Overexpression of Rab3d cDNA in non-invasive MCF-7 cells induces an epithelial to mesenchymal transition (EMT), mediated by activation of AKT/Glycogen synthase kinase 3 beta (GSK3β)/Snail signaling. These effects can be reversed by siRNA-mediated knockdown (KD) of Rab3d in the aggressive triple-negative breast cancer cell line MDA-MB-231, reducing both signaling and expression of EMT markers [18]. One mechanism by which Rab proteins regulate these signaling cascades is through the trafficking of receptor proteins. Rab35 directs tumorigenesis through the synergistic interaction between Rab-driven membrane trafficking and activation of oncogenic signaling [19]. Rab35 activates the phosphatidylinositol-3-kinase/protein kinase B (PI3K/AKT) signaling pathway at lysosome-associated membrane protein 2 (LAMP2)-positive endomembranes in HEK293E cells, by regulating the internalization of platelet-derived growth factor receptor alpha (PDGFRα) [19]. Importantly, the constitutively active form of Rab35 (Rab35Q67L) exerts this effect in a ligand-independent manner. Two naturally occurring gain-of-function mutations observed in tumors, also constitutively activate AKT signaling suggesting that Rab35 mutants enable tumor cells to survive in the absence of growth factor signals.

In addition to activating growth-signaling pathways, Rab proteins are involved in the mediation of other processes associated with tumorigenesis and tumor progression. Notably, Rab5 is essential for hypoxia-driven cell migration and invasion in A549 lung adenocarcinoma cells [20], and induces hypoxia-driven metastasis in non-invasive B16-F10 mouse melanoma cells [20]. Rab25 is another Rab frequently amplified in cancer contributing to the progression of breast and ovarian cancer [21,22]. Specifically, Rab25 mediates invasive migratory phenotypes by directing the localization of integrin-recycling vesicles to the plasma membrane via the association with α5β1 integrins, resulting in increased cell motility in A2780 human ovarian cancer cells [23].

Although less common, Rab proteins are known to inhibit tumor initiation and progression and thus act as tumor suppressive factors. Rab17 suppresses cell proliferation and migration of hepatocellular carcinoma cells in vitro and in vivo while reducing the growth of tumor xenografts in an extracellular signal-regulated kinase (ERK) signaling-dependent manner [24]. Similarly, Rab37 represents an anti-metastatic factor in non-small cell lung cancer (NSCLC) by inhibiting matrix metalloproteinase 9 (MMP9) activity both in vitro and in vivo [25,26]. 

Interestingly, Rab25 can act as a tumor suppressor in claudin-low breast cancers and in bowel epithelial carcinoma cells [27,28]. In addition, Rab25 displays anti-invasive and anti-tumorigenic properties by down-regulation of the focal adhesion kinase (FAK)-rapidly accelerated fibrosarcoma (Raf)-mitogen activated protein kinase/ ERK kinase (MEK) 1/2-ERK (FAK-Raf-MEK1/2-ERK) signaling pathway when overexpressed in EC18 and EC109 esophageal squamous cell carcinoma cell lines [29]. 

## 4. Regulation of Apoptosis by Rab Proteins

**Figure 3:**
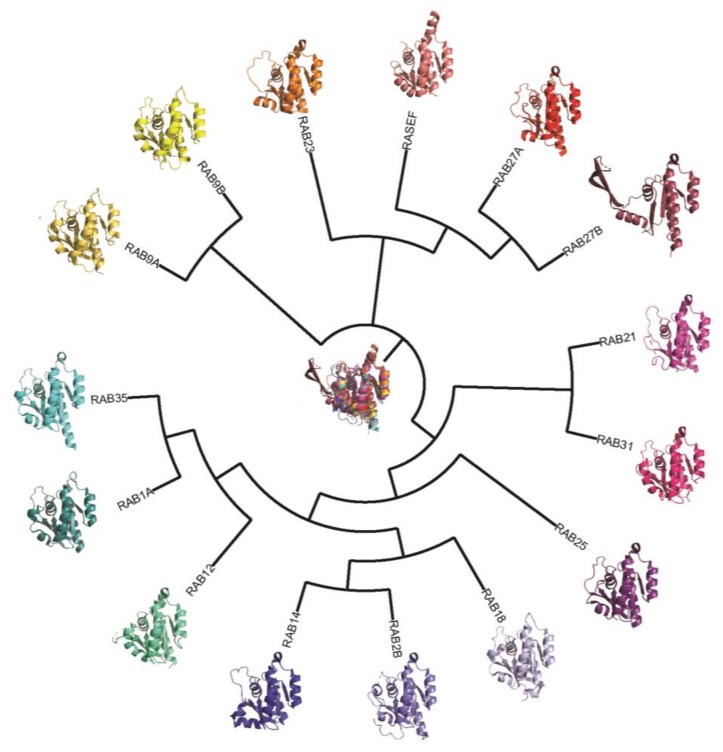
Phylogenetic tree of selected human Rab GTPases involved in apoptosis. Structures and phylogenetic trees of selected Rab proteins discussed in this review. Structure images were produced using Pymol from the protein data bank (PDB) structures (2F7S, 20CB, 4QXa, 1Z22, 2P5S, 6HUF, 1YZT, 2FGS, 2A5J, 1Z0F, 1X3S, 2IL1, 3KTL).

Evading apoptosis is a key hallmark of cancer, which enables cancerous lesions to proliferate and survive without being eliminated. The focus of this section is to review Rab proteins that have pro- and anti-apoptotic functions (Figure 3) and thus hold the potential to be exploited therapeutically.

### 4.1. Overview of Apoptotic Signaling Pathways 

**Figure 4:**
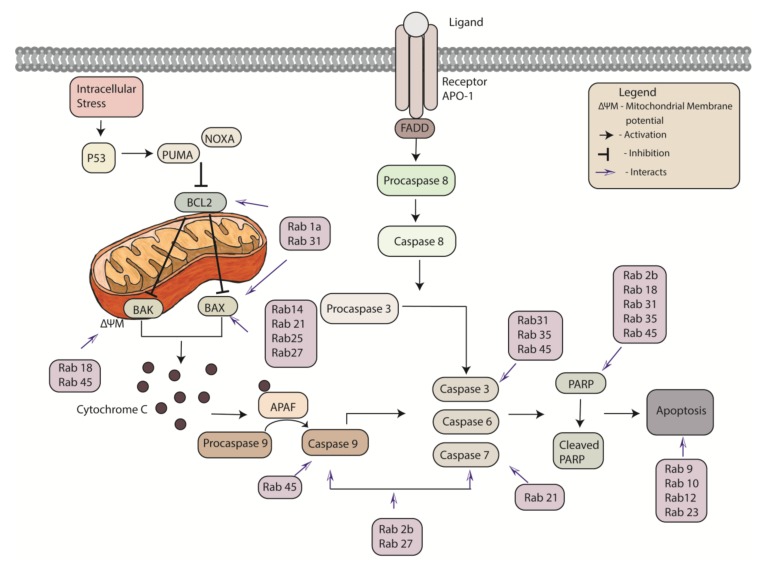
Apoptotic signaling pathways with the reported interaction of Rab proteins. The intrinsic (mitochondrial) pathway is initiated by an internal stress and triggers the induction of apoptotic B cell lymphoma protein 2 (BCL-2) proteins. The BCL-2 family consists of pro-apoptotic BCL-2 homologous antagonist killer (BAK), BCL-2 associated X protein (BAX) and anti-apoptotic BCL-2. Mitochondrial membrane potential (ΔΨM) releases apoptotic proteins into the cytosol. Cytochrome c forms an apoptosome with apoptotic protease-activating factor-1 (APAF-1) converting procaspase-9 into caspase 9. In the extrinsic pathway an external stimulus activates cell surface receptors such as apoptosis antigen-1 (APO-1). The activation enables the recruitment of the adapter protein, Fas-associated protein with death domain (FADD) and activates procaspase 8. Both pathways lead to the activation of execution cysteinyl aspartic acid-proteases (caspases) including initiator caspases (2,8,9,10) and executioner caspases (3,6,7). Initiator caspases bind to proteins such as FADD and APAF-1. Executioner caspases activate cytoplasmic endonuclease and proteases and cleave various substrates such as poly (ADP-ribose) polymerase (PARP).

There are two major pathways regulating apoptotic cell death: the intrinsic and extrinsic pathways (Figure 4). The specificity of these pathways ensure that apoptosis is not initiated randomly, which could be highly detrimental to cells and tissues. Apoptosis is instead triggered upon precise signal induction. 

The intrinsic pathway, which is mediated by the mitochondria, is initiated by internal stimuli such as hypoxia, DNA damage, endoplasmic reticulum and metabolic stress [30]. These receptor-independent stimuli affect mitochondrial membrane integrity by triggering the induction of the apoptotic B cell lymphoma protein 2 (BCL-2) family of proteins [31]. The BCL-2 family is large and diverse, and consists of (1) the pro-apoptotic BCL-2 homologous antagonist killer (BAK) and BCL-2 associated X protein (BAX), (2) the anti-apoptotic BCL-2, BCL-XL, BCL-W MCL-1 and A1/BFL-1 and (3) the pro-apoptotic BH3 only proteins [32]. The BH3 only proteins can be further classified into ‘activating’ such as BH3-interacting domain death agonist (BID) and BCL-2 like protein 11 (BIM), or ‘sensitizing’ such as BCL-2 associated agonist of cell death (BAD) and BCL-2 interacting killer (BIK). 

BAK and BAX oligomerize to form pores resulting in mitochondrial membrane permeabilization, enabling the release of apoptotic proteins such as cytochrome c into the cytosol. Cytochrome c forms an apoptosome with apoptotic protease activating factor (APAF), which activates procaspase-9 [33]. The activating BH3-only proteins directly initiate apoptosis by inducing BAX and BAK oligomerization. Whereas the sensitizing BH3-only proteins prevent the inhibition of the activating BH3-only proteins by binding to the anti-apoptotic BCL-2 proteins. The BCL-2 proteins prevent apoptosis by sequestering BAK, BAX and the BH3-only proteins. 

The extrinsic pathway, also known as the death receptor pathway, requires an external receptor-mediated stimuli to stimulate apoptosis. The tumor necrosis factor (TNF) receptor superfamily member, apoptosis antigen-1 (APO-1) also known as Fas is an example of a well characterized death receptor [34,35]. Upon ligand binding, the receptor activates cysteinyl aspartic acid-proteases (caspases) resulting in fragmentation of substrates and consequently apoptosis [30]. 

Both intrinsic and extrinsic pathways lead to the activation of execution caspases. Caspases are highly specific enzymes, which catalyze the proteolysis of selected substrates. Several caspases, including initiator caspases (2,8,9,10) and executioner caspases (3,6,7) play significant roles in apoptosis [31,36]. Initiator caspases, as the name suggests, initiates the cascade through the binding of proteins such as fas-associated death domain (FADD) and apoptotic protease activating factor-1 (APAF-1). Executioner caspases activate cytoplasmic endonuclease and proteases, degrading nuclear and cytoskeletal proteins, respectively. Such executioner caspases cleave various substrates such as poly (ADP-ribose) polymerase (PARP) and cytokeratins (e.g., cytokeratin-18), which brings about many biochemical and cellular changes such as inter-nucleosomal DNA fragmentation, shrinkage of the cell and the dense cytoplasm observed in apoptotic cells [37,38]. Caspase-3 is a dominant factor among executioner caspases, given its involvement in important apoptotic events such as DNA and nuclear fragmentation, plasma membrane and cytoplasmic blebbing, and cleavage of caspase substrates such as α-Fodrin [39].

### 4.2. Rabs Involved in the Regulation of Apoptotic Proteins via Signaling Pathways 

**Figure 5:**
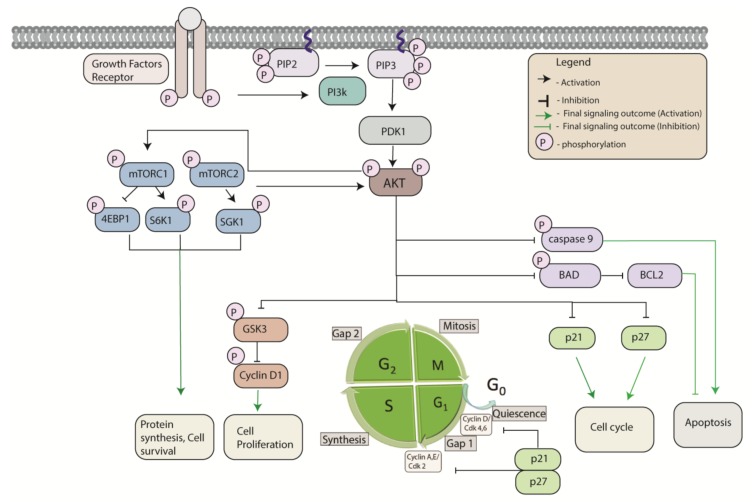
Schematic representation of the phosphatidylinositol 3-kinase (PI3K) signaling pathway and signaling outputs. Conversion of phosphatidylinositol (4,5)-bisphosphate (PIP2) into phosphatidylinositol (3,4,5)-trisphosphate (PIP3), activates phosphatidylinositol-dependent kinase-1 (PDK1) resulting in phosphorylated AKT. Activation of mammalian target of rapamycin complex (mTORC) 1/2 results in phosphorylation of eukaryotic translation initiation factor 4e-binding protein1 (4EBP1)/ribosomal protein S6 kinase 1 (S6K1)/ serum and glucocorticoid-induced protein kinase (SGK). Glycogen synthase kinase 3 (GSK3) inhibits S phase specific cyclin D1 levels, regulating cell proliferation. AKT inhibits cyclin-dependent kinase (CDK) inhibitors, p21 and p27 promoting cell cycle progression. AKT inhibits the activation of caspase 9 and BCL-2 associated agonist of cell death (BAD), resulting in the inhibition of apoptosis.

Multiple works have established the role of the phosophatidylinositol 3-kinase (PI3K) signaling pathway in promoting cell proliferation, survival and the prevention of apoptosis [40,41]. Active PI3K converts phosphatidylinositol (4,5)-bisphosphate (PIP2) into phosphatidylinositol (3,4,5)-trisphosphate (PIP3), resulting in the recruitment of phosphatidylinositol-dependent kinase-1 (PDK1) to the plasma membrane where it can phosphorylate and activate AKT [42]. Active AKT phosphorylates multiple downstream substrates such as forkhead box O1 and O3 (FOXO1/3), glycogen synthase kinase 3 (GSK3) and tuberous sclerosis complex 2/mammalian target of rapamycin complex 1 (TSC2/mTORC1) that have significant roles in growth, proliferation, survival, and apoptosis (Figure 5). AKT inhibits apoptosis by several mechanisms. AKT phosphorylates and inhibits the catalytic activity of caspase-9, inhibiting apoptosis [43]. AKT also inhibits apoptosis by phosphorylating BAD, thereby preventing BAD from sequestering BCL-2. Free BCL-2 is then able to inhibit the pro-apoptotic BAK and BAX proteins [44]. The AKT substrate GSK3 also impacts apoptosis through regulation of cell cycle progression. Specifically, GSK3 inhibits cyclin D1 levels, which downregulates cell proliferation [45]. Activated AKT inhibits the cyclin-dependent kinase (CDK) inhibitors, p21 and p27 enhancing cell cycle progression [46]. In the sections below we have highlighted the role of selected Rab proteins that are implicated in cancer and regulation of apoptosis through direct or indirect modulation of PI3K/AKT and other signaling pathways.

#### 4.2.1. Rab25 

Multiple clinical findings support the role of Rab25 as a prognostic marker for patients with several types of cancers such as breast, ovarian and renal cancer [47]. Furthermore, Rab25 mRNA is increased in multiple other cancer types including ovarian, prostate, bladder, breast and liver cancers making it a highly relevant target for potential cancer therapies. Rab25 modulates the PI3K/AKT pathway and controls proliferation and apoptosis [48,49,50,51,52]. Rab25 directly interacts with AKT and Rab25 levels are positively correlated with activated AKT levels in ovarian tumor samples [53]. In vitro, ectopic overexpression of Rab25 reduces the levels of the pro-apoptotic BAX and BAK proteins in A2780 ovarian cells while KD of Rab25 exerts opposing effects, increasing the levels of BAX and BAK [21,53]. Additionally, Rab25 plays a role in cancer cell survival through the inhibition of nutrient-stress induced apoptosis and autophagy [52]. AKT activation by Rab25 leads to the inhibition of GSK3 and increased levels of glucose uptake, allowing cells to survive longer under nutrient stress [52]. 

#### 4.2.2. Rab31 

Established as a breast cancer marker with good prognostic value, the overexpression of Rab31 is associated with estrogen receptor positive (ER+) breast cancer. Indeed, overexpression of Rab31 promotes the shift from an invasive to proliferative phenotype in breast cancer cells and in xenograft mouse models [54,55]. It is postulated that the overexpression of Rab31 in ER+ breast cancer is due to the ER responsive element in the Rab31 promoter region that results in an estrogen-induced activation of gene transcription [56]. Beyond breast cancer, Rab31 regulates the PI3K/AKT axis in hepatocellular carcinoma (HCC) cells, with KD of Rab31 increasing caspase 3 and 7 activity in human MHCC97 HCC cells. Further in vitro studies indicate that silencing of Rab31 in HCC cells reduces PI3K activation, P101 expression, phosphorylation of AKT and also decreases the BCL-2/BAX expression ratios favoring activation over inhibition of apoptosis [57]. Similar studies in glioblastoma, cervical and gastric cancer confirm the role of Rab31 in regulating apoptosis with overexpression of Rab31 correlated with decreased expression of BAX, cleaved caspase 3 and PARP, concomitant with increased expression of BCL-2 [58]. Similarly in gastric cancer cells, KD of Rab31 by siRNA reduced expression of the BCL-2, Hedgehog (Hh) signaling transcript GLI1 while increasing BAX protein levels [59]. 

#### 4.2.3. Rab35

Rab35 is localized predominantly to both the plasma membrane and endosomes [19] and has been implicated in regulating a variety of processes including vesicle trafficking, endosome dynamics, cytokinesis, signaling and actin remodeling [22,60,61]. In adipocytes, Rab35 regulates trafficking of the glucose transporter isoform 4 (GLUT4) in response to insulin. Recently, it was found that Rab35 mutants can act as oncogenes. The GTPase-deficient Rab35 mutant (Rab35Q67L) activates the PI3K signaling pathway independent of growth factor stimulation and suppresses apoptosis in human embryonic kidney HEK293E cells [19]. GTP-bound Rab35 activates PI3K/AKT signaling in growth factor deprived conditions, concurrent with increased phosphorylation of AKT and FOXO1/3A. Similarly, Rab35 activating mutations (A151T and F161L) found in human cancers (lung, uterus and lymphoid tissues) also upregulate PI3K/AKT signaling. Ectopic expression of wild type Rab35 increases the levels of cleaved apoptotic proteins PARP and caspase 3 and increases cell death, while expression of Rab35 mutants demonstrate significantly lower levels of the cleaved apoptotic proteins and improved cell viability [19].

#### 4.2.4. Rab14

Similar to Rab35, Rab14 is implicated in the trafficking of GLUT4 in adipocytes and regulates AKT signaling. Rab14 is also localized predominantly in the Golgi/Trans Golgi Network (TGN) and in early endosomes (EE) [62]. Optimal Rab14 expression is required for appropriate regulation of EE to TGN trafficking, as retention of GLUT4 is observed in early-endosomal compartments both with depletion of Rab14 or by overexpression of the Rab14Q70L constitutively active mutant in 3T3-L1 pre-adipocytes [63]. Rab14 silencing drives apoptosis in SGC-7901 and BGC-823 gastric cancer lines and this is associated with reduced AKT activation and cell proliferation and increased expression of BAX apoptotic proteins [64]. Conversely, overexpression of Rab14 cDNA specifically decreases the expression of the pro-apoptotic protein BAX in SGC-7901 cells, and KD of Rab14 (shRab14) in BGC-823 cells elevates BAX protein expression consequently promoting apoptosis [64]. 

#### 4.2.5. Rab2b

Rab2b is another Rab protein mainly localized in the Golgi and mediates protein transport from the ER to the Golgi complex [65]. Rab2b also links AKT activation and apoptosis in pancreatic cancer. miR-448-induced down-regulation of Rab2b in PANC-1 pancreatic cells results in silencing of the AKT/mTOR signaling pathway and induces apoptosis with a significant increase in caspase-3, caspase-9 and PARP [66]. Downregulation of Rab2b levels causes G0/G1 cell cycle arrest and promotes apoptosis in PANC-1 cells and alters the expression of cell cycle regulators, including increasing of cyclin D1 and decreasing of p21 and p27. 

### 4.3. Rabs that Regulate Mitochondrial Membrane Potential

Rab18 is a key regulator of neural development by modulating neuronal growth and migration in the cortex during development [67]. Inactivating mutations in Rab18 have been discovered in patients with Warburg Micro syndrome, an autosomal recessive genetic disorder that can produce severe mental retardation. Apart from the known function of vesicle trafficking in neurons, Rab18 also contributes to intracellular lipid homeostasis by modulating lipid droplet growth and maturation [68]. A recent study demonstrated that Rab18 conveyed resistance to cisplatin-induced apoptosis in gastric cancer cells. Cisplatin causes the production of damaging reactive oxygen species (ROS) resulting in reduction of the mitochondrial membrane potential (MMP) in SNU-1 and AGS gastric cancer cells, triggering apoptosis [69]. Rab18 KD in cisplatin-treated cells causes the downregulation of MMP and release of cytochrome c into the cytosol, which in turn activates caspases and PARP, inducing apoptosis. In contrast, the overexpression of Rab18 prevents downregulation of MMP in cisplatin-treated cells rendering them resistant to apoptosis. Over-expression of Rab18 also increases the levels of the mitochondria-localized protein, survivin, which negatively regulates ROS in the mitochondria inhibiting apoptosis. However, Rab18 does not modulate protein levels of survivin’s own regulator, aurora-B, suggesting that the mechanism of Rab18 inhibition of apoptosis is by maintenance of MMP through upregulation of survivin [69]. 

Rab45 represents a novel diagnostic biomarker and a potential therapeutic target for lung cancer [70,71]. KD of Rab45 by siRNA results in a significant decrease in the cell growth of NSCLC [70]. Conversely, Rab45 has also been implicated as a tumor suppressor. Overexpression of Rab45 cDNA induces apoptosis in human chronic myelogenous leukemia (CML) cell lines (K562, Meg01 and SHG3) [72]. In this case, apoptosis is induced through the loss of MMP, leading to the activation and cleavage of PARP, caspase 3 and 9, and a decreased level of expression of inhibitor of apoptosis proteins (IAP) c-IAP1 and c-IAP2, and Survivin, but not BCL-2. This suggests that Rab18 suppresses apoptosis through the IAP and not the BCL-2 family [72]. 

### 4.4. Regulation of Apoptosis through Production of Intracellular Stress

Rab1a represents an important therapeutic target as it has been implicated in the pathogenesis of several human diseases such as cardiomyopathy, Parkinson’s disease and cancer. Rab1a regulates several cell-signaling pathways, such as mTOR and Notch [73,74,75]. A study identified Rab1a as an oncogene in colorectal carcinoma, promoting oncogenesis by direct interaction with mTORC1 resulting in hyper activation of the pathway [16]. Beyond cancer, Rab1a proteins play a causal role in the pathogenesis of several human diseases such as cardiomyopathy and Parkinson’s disease [73,74,75]. As a Golgi-localized Rab, regulating trafficking from the endoplasmic reticulum (ER) to the Golgi apparatus [75,76], Rab1a dysregulation induces apoptosis in cells through the induction of cellular stress. Suppression of Rab1a by miR-15b-5p or by shRNA induces apoptosis and reduces growth and proliferation in both SMMC-7721 and Hep3B human HCC cells, both in vitro and in vivo [77]. Inhibition of protein trafficking from the ER to the Golgi by Rab1a KD results in an accumulation of proteins in the ER causing ER stress and activating apoptotic programs as evidenced by increased levels of BAX expression and decreased levels of BCL-2 [77].

### 4.5. Rabs Regulating Cell Cycle Progression

A number of studies have reported that manipulation of the cell cycle has the potential to prevent or induce apoptotic responses [78,79]. Several proteins such as cyclins and cyclin-dependent kinases (CDKs) coordinate cell division. As discussed above, AKT regulates the CDK inhibitors p21 and p27 altering activity of CDK 2, 4 and 6 [46]. Unsurprisingly, several Rab proteins regulating AKT-mediated apoptosis also orchestrate cell cycle progression, notably Rab2b and Rab18. Other Rabs that regulate apoptosis through cell cycle progression (Rab13 [80], Rab21 [81] and Rab27 [82]) or by means of alternative molecular mechanisms (Rab 9 [83], Rab10 [84], Rab12 [85] and Rab23 [86]) are summarized in Table 1.

## 5. Therapeutic Strategies to Modulate Apoptosis 

Given the broad role of Rab-GTPases in promoting oncogenesis and tumor progression through multiple molecular mechanisms including the suppression of apoptosis, the development of treatments that target Rabs is an alluring prospect. However, given the high structural and sequence similarity that exists between Rab proteins and their isoforms, the challenge remains in designing drugs that can specifically target the desired Rab protein without impacting the Rabs required for “house-keeping” functions of the cell. Development of small molecule inhibitors blocking the interactions between Rab and their effectors, [96] could prove a viable approach to enhancing the selectivity of Rab targeting to “edit” the underpinning Rab-signaling network, a strategy that has been successfully exploited with other difficult drugs and highly pleiotropic targets such as C-MYC and RAS proteins [97].

Other promising treatment approaches are based on small molecules directed against downstream Rab targets, such as the BCL-2 family of proteins, p53, IAPS, survivin and targeting caspases, which have demonstrated great potential for the elimination of cancer cells [98]. This section focuses on the development of treatment strategies targeting apoptotic factors downstream of the Rab factors, with a particular focus on the BCL-2 family of proteins and caspases. We acknowledge that this section is extensive and we direct readers to additional reviews on apoptosis based treatment approaches by U Fischer and K Schulze-Osthoff [99], R. Wong [98] and a review primarily focusing on BCL-2 inhibitors by Min H.Kang and C.Patrick Reynolds [100].

### 5.1. Treatment Approaches Targeting the Anti-Apoptotic BCL-2 Family of Proteins

There are several treatment strategies targeting the BCL-2 family of proteins, including small molecule inhibitors [98]. Some of these inhibitors alter either gene or protein expression of apoptotic proteins such as sodium butyrate and flavopiridol, respectively. In contrast, other inhibitors directly interact with apoptotic proteins such as oblimersen sodium, ABT-263 (ABT-737 analogue) and gossypol, which inhibit anti-apoptotic BCL-2 proteins driving the cells towards apoptosis. These drugs have entered clinical trials for treatments of various cancers such as chronic lymphocytic leukemia (CLL), melanoma and prostate cancer [100,101,102,103]. 

The small molecule ABT-737 is a BH3 mimetic, which does not directly activate the pro-apoptotic BH3 domain proteins BAK and BAX. It instead exerts its pro-apoptotic function in an indirect manner similar to the ‘sensitizer’ BH3-only proteins, BAD and BIK, as mentioned in Section 4.1. ABT-737 inhibits several of the anti-apoptotic BCL-2 proteins preventing them from sequestering BAK and BAX and eventually inducing malignant cells to undergo apoptosis [98,100]. There have been several pre-clinical studies investigating the therapeutic efficacy of ABT-737 in cancers, including small cell lung cancer (SCLC), CLL and multiple myeloma (MM) [100]. CLL cells demonstrated high sensitivity to ABT-737 with induction of apoptosis initiated within a period of 48 hours after treatment and a median effective concentration (EC_50_) of 4.5 ± 2.2 nM [104].

Combination treatment using ABT-737 has been shown to increase therapeutic outcomes. For example, ABT-737 enhances the response to radiation and several chemotherapy agents in SCLC cell models. ABT-737 also sensitizes A549 NSCLC cells to paclitaxel, enhancing the cytotoxicity by a factor of four [105] and combination treatment of ABT-737 with dexamethasone (Dex) has been shown to enhance the anti-cancer effects in MM cells compared to ABT-737 alone [106]. 

Despite its ability to inhibit BCL-2, BCL-XL and BCL-w with high affinity, ABT-737 shows a relatively low affinity to myeloid cell leukemia sequence 1 (MCL-1) [105]. Several reports associate resistance to ABT-737 with high expression levels of MCL-1 and it has therefore been proposed that the inhibition of MCL-1 increases the anti-cancer effect of ABT-737 [107,108].

The combination of cyclin-dependent kinase inhibitors that affect gene or protein expression such as flavopiridol and fenretinide with ABT-737 demonstrated pharmacological synergistic interactions by inactivating MCL-1. Gossypol (AT-101, Ascenta), an inhibitor of BCL (BCL-2, BCL-XL, BCL-w and MCL-1) proteins, is currently in phase II clinical trials in SCLC patients with recurrent chemotherapy and also in hormone-refractory prostate cancer (in combination with docetaxel) [109,110]. AT-101 is a BH3 mimetic administered orally, inhibiting the hetero-dimerization of BCL-XL, BCL-w and MCL-1 with pro-apoptotic BH3-only proteins such as NOXA and PUMA, altering the ratio of pro-apoptotic and anti-apoptotic proteins to induce apoptosis [100,109].

The lack of oral bioavailability for ABT-737 was a main limitation for administration in patients, which led to the development of ABT-263, an ABT-737 analogue [111]. The second-generation compound, ABT-263 displays a similar mechanism to that of ABT-737 (a BAD-like BH3 mimetic) and its oral bioavailability provides dosing flexibility to enhance efficacy as a single agent or in combination with other chemotherapeutics [111]. ABT-263 underwent phase I trials in patients with SCLC and CML and showed promising results in SCLC patients [112]. ABT-263 was recently in Phase IIa trials in patients with refractory or relapsed lymphoid malignancies [113]. Despite the potency of ABT-263, toxic side effects such as thrombocytopenia were observed. This occurred due to inhibition of BCL-XL, which serves as the primary survival factor in platelets. This, prompted the development of new compounds, such as ABT-199, which is a BCL-2 selective inhibitor with a lower binding affinity for BCL-XL [114]. ABT-199 demonstrates significantly less platelet cytotoxicity compared to ABT-293 making it a promising compound that is currently in phase II trials for relapsed and refractory CLL patients and it is also actively being investigated in multiple myeloma studies [115]. 

MCL-1 is another attractive therapeutic target as it is highly expressed in human cancers. MCL-1 inhibitors primarily use the BAD-like BH3 mimetic mechanism. Many of these small molecule inhibitors with a high affinity for MCL-1 have been further developed and have entered clinical trials. AT-101 has been administered in combination with ABT-737 representing the first small molecule with anti MCL-1 activity investigated in clinical trials [109]. Additionally, a number of MCL-1 targeting compounds (MIK665, S64315, AMG176, AZD5991) have entered phase I clinical trials as a single agent for lymphoma, myeloma and hematologic malignancies [116].

Apart from small molecule drugs, silencing anti-apoptotic proteins belonging to the BCL-2 family of proteins is another therapeutic strategy [100]. One study investigated the use of siRNAs as a treatment in pancreatic carcinoma. siRNAs were intraperitoneally administered in pancreatic xenografts, in male nude mice, daily over a period of 24 days and displayed quick distribution to all organs [117]. This showed that with the use of the BCL-2 specific siRNA the expression of target genes in vitro and in vivo were inhibited, consequently displaying pro-apoptotic properties [117].

### 5.2. Treatment Approaches Based on Targeting Caspases 

Research is also being carried out to enable synthetic activation of caspases exploiting caspase- based drug therapy. For instance, peptides containing the arginine-glycine-aspartate motif, known as small molecule caspase activators, are capable of directly activating pro-caspase 3 thereby enabling apoptosis [118]; these caspase activators aid in increasing the drug sensitivity of cancer cells. One study demonstrated an increase in efficacy of the drug doxorubicin when coupled with an intravenously delivered arginine-glycine-aspartate containing cyclic peptide, in human breast cancer xenografts [119]. Caspase-based gene therapy is another approach of interest to induce apoptosis. An example is the recombinant adenovirus containing immunocaspase 3, which displays inhibitory effects on the proliferation of alpha-fetoprotein-producing human hepatocellular carcinoma cells [120].

## 6. Conclusions

Accumulating evidence shedding light on the effects that dysregulated apoptosis has on human diseases, particularly in cancer, has fueled interest in new therapeutic interventions. The treatment approaches targeting apoptosis that are currently in clinical trials raises questions on the safety and effectiveness of these drugs, in particular the effects they have on normal cells and if the treatment strategies could potentially induce resistance in tumors. Addressing these concerns, it would be beneficial to direct future research to dissecting the molecular mechanisms that enable Rab proteins to fine-tune the regulation of apoptosis in cancer and normal cells. The involvement of Rabs in cancer pathogenesis and in a growing list of diseases enlists them as promising new candidates for targeted therapy. Future approaches could focus on targeting the interaction between Rabs and their cellular cofactors, or use genetic approaches to modify the expression of specific Rabs in specific cellular targets i.e., “Rab-editing”. In this regard, it is essential to comprehend how specific Rabs interact with apoptotic proteins, which could then be optimally exploited to orchestrate efficacious treatment approaches against malignant cells.

## Figures and Tables

**Table 1:** Rab proteins involved in apoptosis.

Rabs	* Genetic Alterations	Localization	Cancer Type	Molecular Function	Ref(s)
Rab1a	Amplification	ER, Golgi	Liver	Inhibition of Rab1a (shRab1a) induces apoptosis in HCC	[76,77]
Rab2b	Amplification	Golgi	Pancreas	Inhibition of Rab2b (by miR-448) induces apoptosis in pancreatic cancer cells	[65,66]
Rab9	Deletion/ Amplification	Late endosomes	Breast	Inhibition of Rab9 (siRab9) induces apoptosis	[83,87]
Rab10	Amplification	ER	Liver	Inhibition of Rab10 (shRab10) induces apoptosis in HCC	[84,88]
Rab12	Amplification	Golgi	Gastric	Inhibition of Rab12 (siRab12) promotes apoptosis	[85,89]
Rab13	Amplification		Brain	Inhibition of Rab13 induces apoptosis in glioma cells	[80]
Rab14	Amplification	Golgi/TGN/Early endosomes	Gastric	Inhibition of Rab14 (shRab14) induces apoptosis in gastric cancer cells	[62,64]
Rab18	Amplification	ER	Gastric	Overexpression of Rab18 cDNA inhibits apoptosis through the intrinsic pathway in gastric cancer cells	[69,90]
Rab21	Amplification	Early endosomes	Brain	Inhibition of Rab21 (siRab21) induces apoptosis in glioma cells	[81,91]
Rab23	Amplification	Plasma membrane	Breast	Rab23 elevates breast cancer cell apoptosis	[86,92]
Rab25	Amplification	TGN, Apical recycling endosomes,	Breast	Overexpression of Rab25 cDNA inhibits apoptosis	[21,48]
Rab27a/b	Deletion/ Missense	Endosomal exocytic vesicles	Pancreas, Colorectal	Inhibition of Rab27a/b (siRab27a/b) induces apoptosis in pancreatic cells	[82,93,94]
Rab31	Amplification	Late endosomes, Trans Golgi, TGN	Gastric Liver Brain/ Cervix	Overexpression of Rab31 cDNA inhibits apoptosis in gastric cancer cells Inhibition of Rab31 (siRab31) induces apoptosis in HCC Overexpression of Rab31 cDNA inhibits apoptosis in glioblastoma and cervical cancer cells	[57,58,59,95]
Rab35	Amplification/ Missense mutation	Plasma membrane, Endosomes	Kidney	Overexpression of Rab35 cDNA expression suppresses apoptosis	[19]
Rab45	Missense mutation	Perinuclear region	Leukemia	Overexpression of Rab45 cDNA induces apoptosis	[71,72]

* Genetic alterations (from cBioPortal for cancer genomics) that were the highest across cancers are listed above. HCC: Hepatocellular carcinoma, TGN: Trans Golgi network, ER: Endoplasmic reticulum.
